# Effects of recycling and bonding agent application on bond strength of stainless steel orthodontic brackets

**DOI:** 10.4317/jced.51113

**Published:** 2013-10-01

**Authors:** Faisal I. Bahnasi, Aida NA. Abd-Rahman, Mohame I. Abu-Hassan

**Affiliations:** 1 PhD Candidate, Centre of Studies for Paediatric Dentistry and Orthodontic, Faculty of Dentistry, Universiti Teknologi MARA, Shah Alam, Selangor, Malaysia; 2 Lecturer, Centre of Studies for Paediatric Dentistry and Orthodontic, Faculty of Dentistry, Universiti Teknologi MARA, Shah Alam, Selangor, Malaysia; 3 Dean, Professor, Centre of Studies for Restorative Dentistry, Faculty of Dentistry, Universiti Teknologi MARA, Shah Alam, Selangor, Malaysia

## Abstract

Objectives: 1) to assess different methods of recycling orthodontic brackets, 2) to evaluate Shear Bond Strength (SBS) of (a) new, (b) recycled and (c) repeated recycled stainless steel brackets (i) with and (ii) without bracket base primer. Study Design: A total of 180 extracted human premolar teeth and 180 premolar stainless steel brackets were used. One hundred teeth and 100 brackets were divided into five groups of 20-teeth each. Four methods of recycling orthodontic brackets were used in each of the first four groups while the last one (group V) was used as the control. Groups (I-V) were subjected to shear force within half an hour until the brackets debond. SBS was measured and the method showing the highest SBS was selected. A New group (VI) was recycled twice with the selected method. Six subgroups (1-6) were established; the primer was applied for three sub-groups, and the composite was applied for all brackets. Brackets were subjected to the same shear force, and SBS was measured for all sub-groups. Results: There was a significant difference between the mean SBS of the sandblasting method and the means of SBS of each of the other three methods. There was however, no significant difference between the mean SBS of the new bracket and the mean SBS of recycled bracket using sandblasting. The mean SBS of all sub-groups were more than that recommended by Reynolds (17) in 1975. Brackets with primer showed slightly higher SBS compared to those of brackets without bonding agent. Conclusion: To decrease cost, sandblasted recycled orthodontic brackets can be used as an alternative to new brackets. It is recommended to apply a bonding agent on the bracket base to provide greater bond strength.

** Key words:**Recycled bracket, shear bond strength, sandblasting, stainless steel orthodontic bracket.

## Introduction

- Adhesives

Early bonding systems consist of brackets welded onto bands bonded to enamel with zinc phosphate cement. Currently, it is easier to bond bracket to tooth surface using different adhesive materials. Development of modern adhesive materials has led to the widespread use of bonded attachment in fixed appliances. Composite resin is the most popular orthodontic adhesive because of good bond strength (1). According to Owens and Miller (2) “if bond strength is the primary consideration for choosing an adhesive, the composite resin should be utilized”.These adhesives are used currently in orthodontic treatment to bond brackets to teeth surfaces. To achieve the complex tooth movements during orthodontic therapy, clinician requires a reliable method of attachment to tooth tissue. The method of attachment should allow the delivery of orthodontic forces and should be sufficient to withstand masticatory loads. In addition, the attachment should be easily removed at the end of treatment, and result in minimal hard and soft tissue damage during application (3).

- Orthodontic Brackets

There are different types of orthodontic brackets present in the market. Orthodontic brackets could be manufactured from stainless steel or aesthetic material as: ceramic or plastic. Metal brackets are cheaper than ceramic and easy to be recycled to reuse it again in case of bond failure or repositioning of brackets.

- Bracket Recycling 

Orthodontic bracket bond failure is common during orthodontic treatment. To decrease the cost of orthodontic treatment; any debonded bracket can be recycled by different methods to provide a second alternative to new brackets (4). The previous adhesive material can be removed from bracket mesh by different ways such as: laser, sandblasting, grinding, thermal and chemical methods. If Shear Bond Strength (SBS) of recycled orthodontic bracket is not enough to withstand the occlusal force, bond failure will take place. Multiple orthodontic visits for rebonding of recycled brackets are time and material consuming; this may cost more than replacing the failed bracket with a new one. The main advantage of reused orthodontic brackets is that they are cheaper. However there are disadvantages:

1. Possibility of bracket distortion due to high occlusal force, or during debonding process in case of brackets repositioning (5).

2. Method of recycling process is time consuming.

3. Lower SBS of recycled brackets in some methods such as using direct flame (6).

Er,Cr:YSGG laser was a new method of recycling orthodontic brackets. It was an efficient method in removing adhesive from bracket bases, so the brackets can be used again, with good SBS (7). Laser device is still expensive so it is not easily found in dental clinics. Green stone or dental bur grinding is a simple method of recycling, however, it may provide insufficient SBS rates (6,8). Basudan AM and Al-Emran (8) recommended direct flame as a simple and effective method of recycling brackets. However, Quick et al (6) evaluated that direct flame had a low SBS. Aluminum oxide sandblasting is the most popular method. Different studies indicated that the sandblasting method of recycling achieved enough SBS when compared to new brackets (8-11).

- Repeated recycling

Reusing loose brackets after reconditioning is common; however, in case of second bracket bond failure to the same bracket, the clinician may prefer to use a new one instead of reusing the old one for a second time. Bishara et al. (12) studied the effects of repeated bonding with two different adhesives on the SBS of orthodontic brackets and he found that composite adhesive had a higher SBS than the cyanoacrylate adhesive at the second bonding/debonding sequence but not at the third. Bishara et al. (13) evaluated the effect of repeated bonding on the SBS of orthodontic brackets. He found that the highest values for SBS were obtained after the initial bonding. SBS may decrease or increase after the second debonding. This article was focused on the SBS of new, recycled and repeated recycled stainless steel orthodontic brackets to enamel surfaces using different methods of recycling; as till now no article in the literature focused on repeated recycling using these methods as well as the effect of bonding agent application to bracket base on the SBS of new, recycled and repeated recycle stainless steel orthodontic brackets.

## Material and Methods

A total number of 180 extracted human premolar teeth were collected and all blood and adherent tissues were removed from the teeth. Teeth with caries or other visible defects or restorations were excluded. 180 new stainless steel upper premolar orthodontic brackets (Unitek� Gemini Bracket, Micro-Etch Base, 3M Unitek orthodontic products) were used. Bonding agent (Light Cure Orthodontic Adhesive Primer, 3M Unitek) was applied to paper pad. Composite resin (3M Unitek Transbond� XT Light Cure Composite) was applied on 80 brackets. Brackets were positioned on the paper with gentle pressure and excess adhesive was removed with explorer. Polymerization was carried out using Light-emitting diode (LED) (SDI radii- cal) for 20s (10s for each side mesial and distal). The light intensity of LED is 1800 Mw/cm�. The power of device was calibrated before every cure using a dose meter device (SDI radiometer). The bonded brackets were separated from the paper pad using tweezers with light pressure. Another 20 orthodontic brackets were considered as a control group. All 80 brackets were stored for distilled water for 24h at 37 �C, and then they were subjected to the thermocycling using Automatic Thermocycling Dipping Machine (ATDM T6PD, Zecttron Sdn. Bhd., Malaysia) for 500 cycles in distilled water between 5 �C and 55 �C. The exposure to each bath will be 20s and the transfer time between baths will be 5s. The total number of 80 orthodontic brackets and 80 extracted human premolar teeth were randomly divided into 4 Groups (I-IV). Another 20 new brackets and teeth were considered as a control group (Group V). Group�s brackets (I-IV) were recycled using four methods of recycling.

� Group I 

Sandblasting with 50 ?m aluminum oxide particle powder was done from a distance of 10 mm away from the nozzle of Microetcher (MicroEtcher� Intraoral Sandblaster, Danville Materials, Innovative Dental Products, San Ramon, CA 94583, USA) under the air pressure of 90 PSI for 20-30s depend on the amount of adhesive remaining. The procedure was followed until bonding resin was totally removed from the bracket base and was no longer visible to the naked eye.

� Group II 

Removal of the resin using gold coated carbide bur by high-speed hand piece (350000�370000 rpm, W&H, Austria) with air cooling until most of the residue was removed.

� Group III

Direct flame was applied at the tip of the gas torch flame (GT Prince 3000S Micro Torch/PAT.P., Japan), and then pointed at the bracket base for 5s until the bracket base became red hot, then quenched in water at room temperature, and dried in an air stream.

� Group IV 

The brackets were immersed in 95% sulphuric acid for 10 min until the adhesive become easily removed from bracket base.

- Teeth sample preparation

The teeth were embedded horizontally in die stone in a plastic ring. The buccal surface of the teeth were cleaned with a non-fluoridated prophylaxis powder with a rubber cup at low speed hand piece for 15s, rinsed with water for 10s, and dried with a light/brief stream of oil-free compressed air. The teeth were etched with 35% phosphoric acid gel for 15s (Unitek� Etching Gel Syringe 35% phosphoric acid by weight, 3M Unitek orthodontic products), rinsed with water for 15s and dried with a light/brief of air (oil and water free). Adhesive Primer was applied to the buccal surface of each tooth, thinned with gentle stream of air. Composite resin was applied to all bracket bases. The brackets were then firmly pressed to teeth surfaces with a plastic instrument and the excess adhesive was removed with an explorer before curing. Polymerization was carried out using LED light to cure both mesial and distal sides for 10s each.

- SBS determination

After photo polymerization, Groups I-V were subjected to a shear force within 30 minutes to simulate the clinical situation with a universal testing machine (Shimadzu Trapezium X, Shimadzu Corporation, Kyoto-Japan) until the bracket debonds with a crosshead speed of 1mm/min. The force in Newton (N) was recorded, and the stress was calculated by dividing the force in Newton/surface area and calculated in MPa.

- Data analysis for Groups I-V 

Data was subjected to statistical analysis to identify significant differences between the means in the SBS of the Groups: I-V. The analysis was carried out using SPSS program (SPSS, Chicago, Illinois, USA.

For group comparison, the single factor variance analysis (ANOVA) was used. The level of significance was established at P < 0.05. The best group from I-IV was selected for repeated recycled procedure. The selection of the group was based on.

1. The greatest significant difference in the mean SBS among all the recycled groups.

2. Simplicity, practicality and availability of the method, in case of no significant difference obtained between the groups� mean SBSs.

Repeated recycled procedure 

20 new brackets were recycled twice with the above selected method using the same ways of bonding and de-bonding. 20 premolar teeth were selected and the brackets were bonded using the same etchant, primer and composite. This group was considered as a new group (Group: VI). Group VI was subjected to the same shear force as before.

- Scanning electron microscopy (SEM) evaluation 

The orthodontic brackets� bases were examined using scanning electron microscope (SEM) (Carl Zeiss SMT model: SUPRA� 40 VP) at magnification 100X and 300X to compare the distortion after recycling procedure with the new brackets� base.

Bracket mesh bonding agent

To measure the effect of adding bonding agent on the orthodontic brackets surfaces, six subgroups [Sub Groups 1-6] (n of each subgroup =20) were established consisting of new, recycled and repeated recycled brackets each with or without application of bonding agent to the brackets surfaces. The same procedure of bonding and debonding were again followed, but in the three subgroups applied with bonding agent, light cure orthodontic adhesive primer.

- Data analysis for Sub Groups 1-6

The force and stress applied during SBS testing were calculated, and the data was subjected to statistical analysis to identify significant differences in means SBS between subgroups. ANOVA was used; the level of significance was established at P < 0.05.

## Results

**Figure 1:**
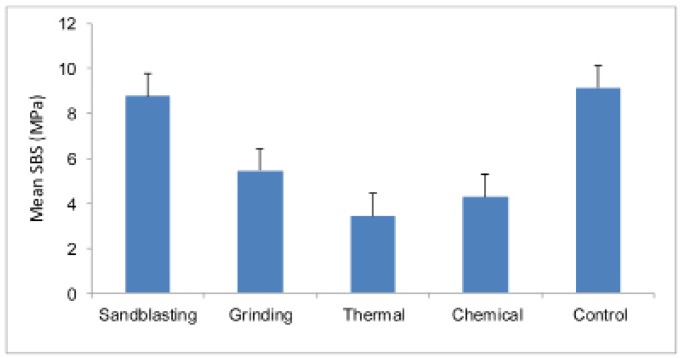
Mean SBS strength of Group I-V.

Based on Table 1 and fig. 1, the p-value is less than 0.001. Thus, at least one pair of means differ significantly. The test of homogeneity of variances gave a p-value of 0.07. Hence equality of variance assumption is met. Based on the Tukey�s post hoc test:

**Table 1:**
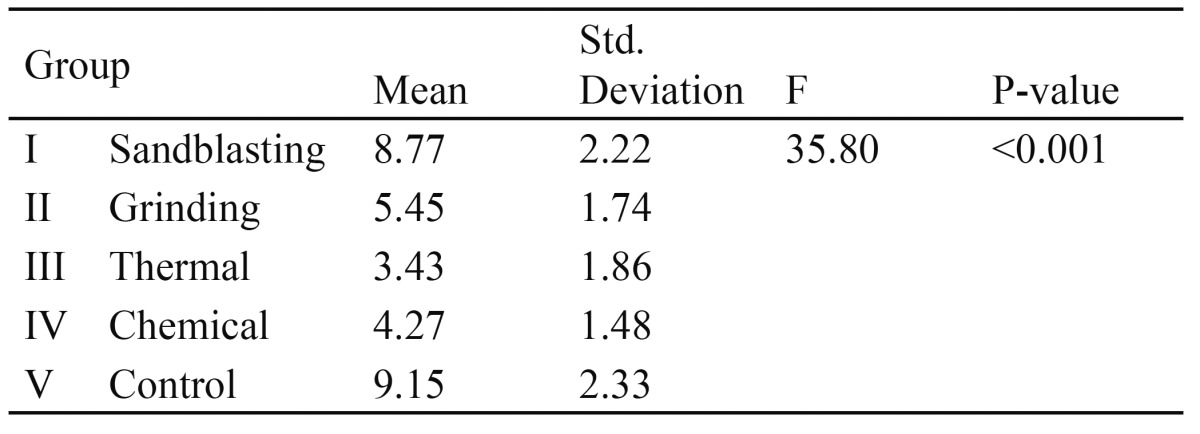
One way ANOVA for groups� I-V

� The mean SBS of Group V (control group) was significantly higher compared to those of Group II (Grinding), Group III (Thermal) and Group IV (Chemical) but not significantly different compared to that of Group I (sandblasting group).

� The mean SBS for the Group I was significantly higher compared to those of Groups II, III and IV.

� The mean SBS for the Grinding group was significantly higher than the Thermal group but not significantly different from the Chemical group.

- Repeated recycling:

Based on the Independent Samples T-Test to compare among Group I (sandblasting group), Group VI (repeated recycle) and Group V (control group) ( Table 2):

**Table 2:**
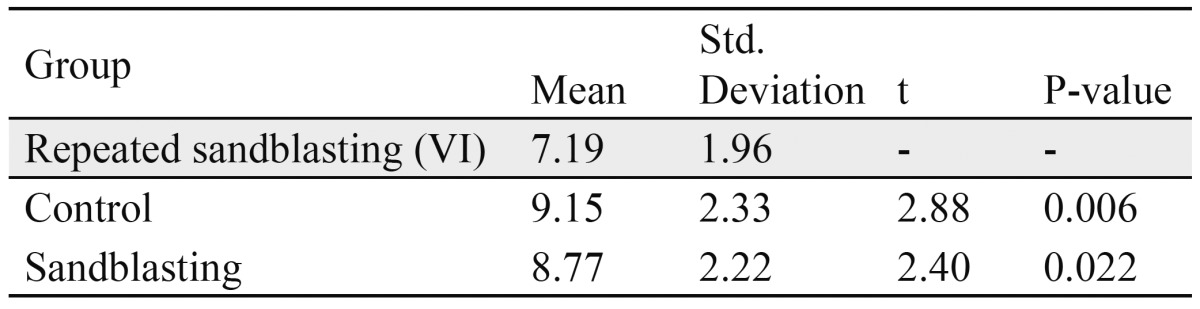
Independent Samples T-Test for group I with groups V and VI respectively.

� The mean SBS for Group VI was significantly lower strength than the mean SBS for the Group V.

� The mean SBS for the Group VI had significantly lower strength than the mean SBS for the Sandblasting group.

**Figure 2:**
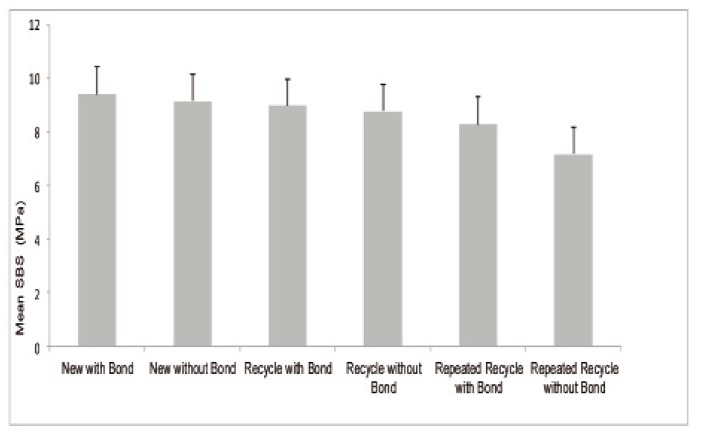
Mean and standard deviation of sub-groups 1-6, the mean SBS of G 1 was (9.41), G 2 (9.15), G 3 (8.98), G 4 (8.77), G 5 (8.29) and G 6 (7.19). There was significantly difference between G 1 and G 6.

For Sub-Groups 1-6, mean SBS and standard deviation were recorded. (Fig. 2). Statistical analysis was perfor-med using one way ANOVA test ( Table 3). Based on  Table 3, the P < 0.05; thus, at least one pair of means differ significantly. The test of homogeneity of variances gave a P-value of 0.858. Hence equality of variance assumption is met. Based on the Tukey�s post hoc test; the mean SBS of Group 1 (new with bond) was significantly higher compared to that of Group 6 (repeated recycle without Bond).

**Table 3:**
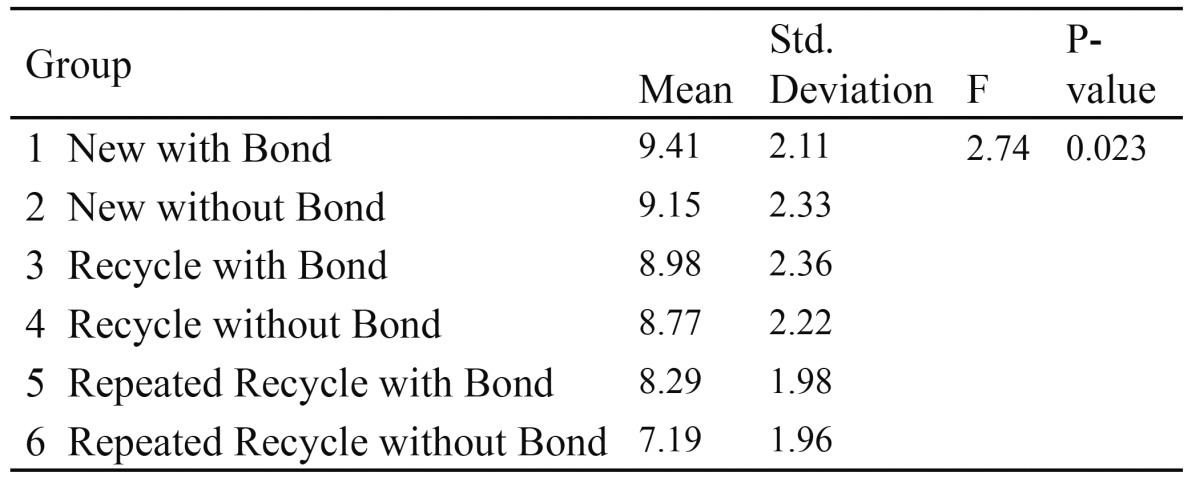
One way ANOVA for sub-groups (1-6).

The Independent Samples T-Test was used to compare between each categories� sub groups 1 and 2; 3 and 4; 5 and 6. There was no significant difference between the means of SBS of the each two groups:

� The mean SBS for Group 1 (new with bond) was not significantly different compared to the mean SBS for Group 2 (new without bond).

� The mean SBS for Group 3 (recycle with Bond) was not significantly different compared to the mean SBS for Group 4(recycle without Bond).

� The mean SBS for Group 5 (repeated recycle with Bond) was not significantly different compared to mean SBS for Group 6 (repeated recycle without Bond).

- SEM evaluation 

**Figure 3:**
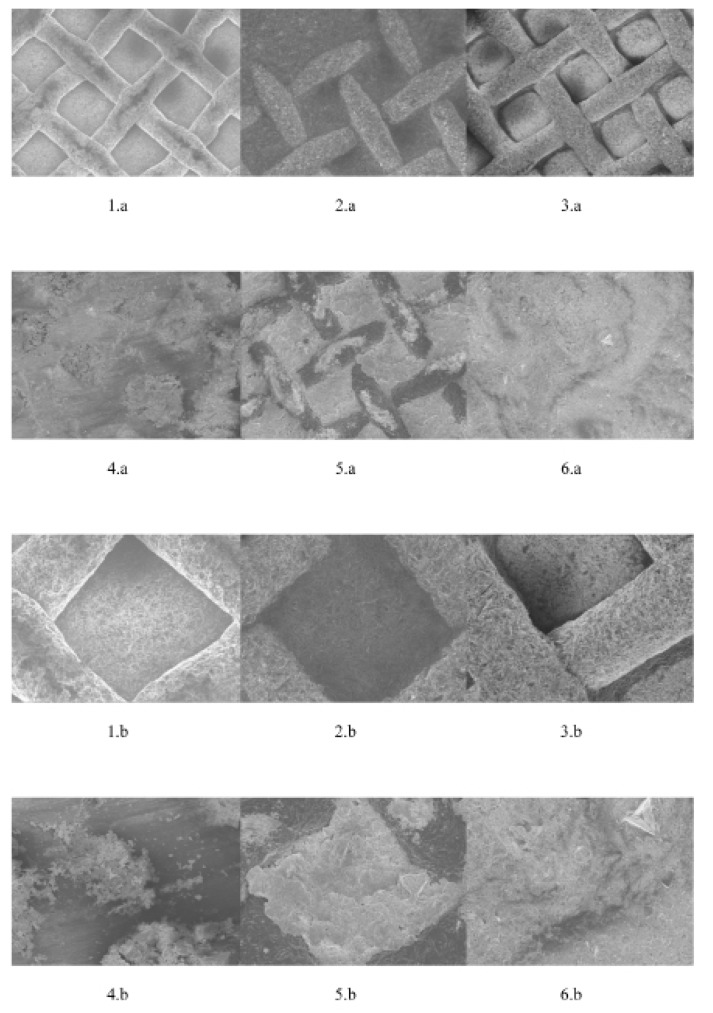
Scanning electron microscopy (SEM) evaluation of the metal orthodontic bracket bases of: new at 100X (1.A) and 300X (1.B), recycled by sandblasting at 100X (2.A) and 300X (2.B), repeated recycled by sandblasting at 100X (3.A) and 300X (3.B), recycled by grinding at 100X (4.A) and 300X (4.B), recycled by thermal at 100X (5.A) and 300X (5.B), recycled by chemical at 100X (6.A) and 300X (6.B).

Fig. 3 showed the evaluation of the orthodontic brackets� bases under the scanning electron microscope at magnification 100X and 300X using different recycling methods.

## Discussion

Different methods of recycling were used, and the sandblasting method (Group I) showed the highest SBS, which is significantly different in comparison to other methods (Groups II-IV). There was no significant difference between new (Group V) and recycled bracket with sandblasting (Group I). The mean SBS of all subgroups were more than that recommended by Reynolds (14) in 1975; (for brackets bonded to teeth to overcome intraoral and orthodontic forces, SBS in the range of 5.9 to 7.8 MPa was required). Although Sub-Group 6 (repeated sandblasting without bond) showed lowest mean SBS, but still enough SBS according to Reynolds (14). There was no significant difference between mean SBS of Sub-Group 2 (New without bond) and that of Sub-Group 4 [Recycle (sandblasting) without bond. The repeated sandblasting group (Sub-Group 6) had significantly lower mean SBS than the control group (Group 2) and sandblasting group (Group 4), hence repeated recycled without bonding agent replication is not effective for clinical use. The current study showed that using recycled-sandblasted brackets may provide a sufficient SBS rate which was in agreement with other studies (8-11). Sandblasted recycled bracket can be used instead of new one, in case of bond failure, to save cost. In case of second time bracket bond failure to the same bracket, the clinician may prefer to use a new one instead of reusing the same bracket for a second time. In current study, the mean SBS of repeated bracket recycling was found to be less than those of new and recycled bracket, unless bonding agent was applied, which provides adequate mean SBS. Bishara et al (13) found that the highest values for SBS were obtained after the initial bonding. SBS may decrease or increase after the second debonding. There being no difference between mean SBS of Sub-Group 1 and 2, between Sub-Group 3 and 4, with/without bonding do not offer any advantage. However, not bonding for the repeated recycle Sub-Group 6 does much to make the SBS very low.

From this study, it can be concluded that:

� Bracket recycling using 50-?m aluminum oxide powder did not affect the SBS of stainless steel brackets and can be used as an alternative to new brackets. This could certainly cut cost.

� Bracket recycling using grinding, thermal or chemical methods showed lower SBS than recommended by Reynolds (14).

� In case of using repeated recycled brackets by sandblasting, the current study shows enhanced bond strength with bonding agent application to the bracket.
